# Development and Initial Validation of the Safety Training Engagement Scale (STE-S)

**DOI:** 10.3390/ejihpe12080070

**Published:** 2022-08-02

**Authors:** Marco Giovanni Mariani, Gerardo Petruzziello, Michela Vignoli, Dina Guglielmi

**Affiliations:** 1 Department of Psychology “Renzo Canestrari”, University of Bologna, 40127 Bologna, Italy; gerardo.petruzziell2@unibo.it; 2 Department of Psychology and Cognitive Science, University of Trento, 38068 Rovereto, Italy; michela.vignoli@unitn.it; 3 Department of Education Studies “Giovanni Maria Bertin”, University of Bologna, 40127 Bologna, Italy; dina.guglielmi@unibo.it

**Keywords:** safety, training, engagement, dedication, absorption, scale validation

## Abstract

Safety training promotes safety at work, in particular through the use of engaging methods. This study introduces a newly developed measure of individual engagement in safety training, and aims to analyze the psychometric proprieties of the scale. The safety training engagement scale (STE) consists of five items pertaining to the trainee’s dedication and absorption in a safety training session. Two studies are carried out to analyze the validity of the scale. The first study focuses on the construct (internal) validity, to examine the scale’s internal consistency and dimensional structure. The second study seeks to provide further evidence for construct validity by testing the external validity of the scale. The sample consists of 913 (study 1) and 133 (study 2) participants in safety training programs in the field of the chemical industry who were invited to fill the STE scale after attending a safety training course. The results provide support to affirm the validity and reliability of the scale. The discussion describes the implication and the limitations of using the STE scale in practical safety training programs, and outlines recommendations for research to improve the scale’s robustness.

## 1. Introduction

In the face of the high number of accidents at work in the EU-28 [1], companies adopted several actions to improve safety at work, such as risk assessment, targeted campaigns, and training programs for risk prevention and safety promotion, especially currently, where many workplaces are high-risk environments for COVID-19 [2]. The interventions on human factors (henceforth, HF) assume critical value in the safety field. Indeed, statistics show that human error is one of the primary causes of failure and accidents in a variety of work contexts, such as the road and transportation [3], chemical and petrochemical [4], marine [5], aviation [6], construction [7] sectors, and engineering and security [8]. In this regard, training is a tool that can improve the impact of HF and occupational health, and can be particularly useful in times such as the COVID-19 situation [9].

The systematic review of Ricci and colleagues [10] asserts that the training processes can improve distinct yet related individual factors such as workers’ knowledge, attitudes, and behaviours concerning safety. Moreover, the same authors underline that the training processes are complex since many elements play a role, such as trainees’/trainers’ characteristics, setting features, session duration, and training methodology [10].

A meta-analytic study by Burke and colleagues [11] found that not all training methods have the same levels of effectiveness. It shows, for instance, that as the methods become more engaging, based on learners’ participation in the training process, the safety courses demonstrate greater effectiveness. Therefore, methods based on passive learning, such as lectures, achieve lower levels of effectiveness than engaging methods with active learning, such as behavioural simulations, where interactions between trainees and trainers are frequent and based on reciprocal feedback [11]. This type of study draws on the principle that an active method improves the trainee engagement, without considering the actual engagement that can be developed. It is important to integrate the perspective of Burke and colleagues [8], focusing on the perceptions, impressions, sensations, and involvement of the trainees who attend a safety course.

Therefore, the present study intends to deepen the exploration of the concept of engaging training methodology from the trainees’ perspective. It proposes a measure that analyses participants’ level of engagement and involvement in a safety course. Based on previous measures of engagement, the scale was developed to help trainers and researchers evaluate the levels of participant engagement in training, specifically in the safety field, in a practical, valid, and reliable way.

This study lies in a scientific field that sees the presence of various instruments for training evaluation (e.g., [12,13,14,15,16]), yet not specifically focused on the engagement and/or validated in the safety training program. Indeed, although many studies emphasize the critical value of engagement in safety training [11,17], and there are scales on trainee reactions (e.g., [12,14]) and various aspects of training from a broader perspective, no measurement tools have been developed and validated for assessing the trainees’ engagement in this field, meaning that a short, valid, and reliable measure designed to assess participants’ engagement in safety training is necessary.

The present study attempts to fill these gaps, and aims to contribute to the literature with a valid, reliable, and practical measurement tool, specifically tested in safety training. Our scale is supposed to add to the training evaluation scholarly field. It is meant to be complementary and integrated, because it provides a training engagement analysis with a brief measure of a few items. It also intends to be specific to the safety context because it is developed and tested in this field.

### 1.1. Theoretical Background

The importance of engagement in safety training comes to light in an essential review by Burke and colleagues [11]. The researchers analyzed the relative effectiveness of different training methods considering three outcomes: (a) safety knowledge, (b) safety performance, and (c) safety and health outcomes (e.g., accidents, illnesses, or injuries). The researchers distinguished three levels of engagement in safety training: (1) least engaging training, which relies on methods such as lectures, videos, pamphlets, and written materials; (2) moderately engaging training, which adopts techniques such as feedback interventions, performance information provided in small groups, and computer-based interactive instruction; and (3) most engaging training methods, where there are hands-on demonstrations associated with behavioural stimulation and active participation of the trainee. The findings underline that the more engaging the training is, the greater the effects on knowledge acquisition are. Furthermore, training will have a more significant impact on reducing adverse safety and health outcomes if the course adopts highly engaging methods. Concerning safety performance [18], the results outlined by the review are mixed, but suggest the effectiveness of more engaging training methods. Overall, Burke and colleagues [11] argued that the most engaging methods in safety training are approximately three times more effective than the least engaging in promoting knowledge and skill acquisition. Robson and colleagues [7] provided a second review where the level of trainees’ engagement categorized training interventions. The review shows mixed findings: on the one hand, if we consider attitudes and health as outcomes, more engaging training methods are more effective than low-engagement ones. On the other hand, when the authors analyzed outcomes, the findings were not in line with the Burke and colleagues’ review [5]. Indeed, the effectiveness of interventions is strong regarding the effect on behaviours, but for health outcomes, the results are not so consistent; however, it should be noted that the latest results are derived from three studies only, two of which consist of very brief interventions. The study by Namian and colleagues [19], conducted in the construction industry and based on interviews and questionnaires with experts and the analysis of empirical data gathered from 51 case projects, presents a picture of findings where high-engagement training methods maximize safety training outcomes.

Having underlined the value of adopting engaging methods in safety training, we now present an analysis of the concept of engaging methods. Burke and colleagues [20] describe a method as more engaging when it incorporates elements of action, dialogue, and reflection, and encourages trainees to infer relations among concepts, events, and actions to develop new ways of thought and action. On a different level, by adopting a subjective perspective, the concept of engagement is broader and extensively studied in organizational psychology. Its relevance rises in the positive psychology that studies human resource strengths, resources, and psychological capacities that can be measured, managed, and effectively developed in the workplace [21]. In this regard, ‘work engagement’ consists of a sense of energetic and effective connection with employees’ work activities, and reflects the ability to deal well with the job demands. The literature [22] defines it as a positive, fulfilling, work-related state of mind experienced by employees, and characterized by vigor, dedication, and absorption. Work engagement is widely studied, and it is considered a result of an energetic process, as opposed to an energy-consuming process leading to burnout [22]. Work engagement is presented as similar to having “flow” (i.e., experiencing a sense of total harmony), but it is more stable than flow, which tends to be a peak experience [23]. Similarly, in learning contexts, situational interest and affective engagement can play important roles, acting as positive motivational states of learners [24,25]. A recent study found that behavioural engagement (defined as effort and perseverance in learning) and emotional engagement (defined as a sense of belonging) significantly predicted academic performance [26].

Recently, Casey and colleagues [27] proposed a specific framework for the safety field that considers training engagement as a combination of pre-training factors (e.g., individual, organizational, and contextual factors) and the training program factors (e.g., learning environment, trainer characteristics, as a trust). Based on the educational psychology literature [13], the authors conceptualized safety training engagement using three-component psychological states (affective, cognitive, and behavioural), which drive the motivation to learn and other training approach behaviours. Affective or emotional engagement refers to a positive mental state concerning the learning task. Cognitive engagement refers to the mental effort invested in the training to think about and attend to the materials, and behavioural engagement as actively participating in the training program.

Eventually, for the definition of the safety training engagement, we followed the definitions provided by Ben-Eliyahu and colleagues [13], and Hallberg and Schaufeli [19]. We consider safety training engagement in terms of dedication and absorption. Dedication reflects how much one is involved in the safety training program, experiencing a sense of significance and enthusiasm (affective state). Absorption pertains to how much the trainee is entirely concentrating on and interested in the safety program (cognitive state), where time passes quickly, and without detaching themselves from the course [28]. So we focus on a mix of affective and cognitive elements [16], analyzed in a synergic way, during a safety training program. This is an additional element that distinguishes the safety training engagement scale (hereafter, STE-S) from the other measures in the literature.

### 1.2. Aims and Hypotheses of the Research

Despite the importance of adopting engaging methods in safety training [11], there are no validated scales in a safety training program that measure trainee engagement with a theoretical perspective that has been described in the previous paragraph, to the best of our knowledge. However, there are many examples in the literature of scales evaluating subjective reactions towards training, which justifies the development of a safety training engagement scale to complement these tools. Morgan and Casper [12] built scales on general trainee reactions in different courses. Ritzmann and colleagues [13] developed the training evaluation inventory, which measures subjective enjoyment, usefulness, difficulty, knowledge gain, and attitude toward training. Grohmann and Kauffeld [14] built a questionnaire for professional training evaluation (Q4TE), which measures short- and long-term training outcomes. Moreover, O’Brien and Toms [15] developed a scale on engagement that focuses only on the peoples’ experiences with technology, from a perspective of the design of interactive systems.

Lastly, the literature presents a specific scale of engagement in training by Ben-Eliyahu and colleagues [16], which provides an analytic and articulate measurement of the construct with satisfactory psychometric proprieties. Yet, it presents some limitations that may affect its application in practical contexts. Indeed, it has not been developed in the safety context, as it was tested among high school students. Moreover, due to its length (it consists of 17 items), it may not be suitable for application in situations where agility is required, such as at the end of a training session. A more agile and less demanding tool is recommended for gathering more reliable data.

Drawing on these bases, our research’s first aim is to develop a short instrument to assess trainees’ engagement in terms of absorption and dedication to the safety training. The study focuses on creating a brief and practical scale (STE-S) that measures this construct in a reliable, valid, and efficient manner, and can be adopted by both researchers and trainers of safety programs. The second aim is the validation of STE-S. Many researchers underlined the relevance of developing valid and usable training evaluation tools [29]. For instance, following this research line, scholars validated some questionnaires: Morgan and Casper [12] studied the factor validity of scales on trainee reactions in different courses. Ritzmann and colleagues [13] analyzed the internal validity of the measures. Grohmann and Kauffeld [14] studied the stability of the questionnaire for professional training evaluation (Q4TE) factor structure.

We wanted to study the psychometric properties by testing the construct in terms of internal and external validity, according to the American Psychological Association [30]. Some essential aspects of internal validity were analyzed, such as dimensionality and reliability, while external validity was tested through criterion-related validity. Given that internal validity focuses primarily on the internal relationships of the test, the first step (Study 1) was to analyze whether the theoretical uni-dimensionality of the scale is supported.

Therefore, our first research questions address the factorial structure and reliability:

**Research question 1**: Do STE-S items construct a uni-dimensional scale?

**Research question 2**: Does STE-S present fair internal consistency?

Furthermore, we aimed to evaluate external validity with a second study. Specifically, the criterion-related validity was tested to assess the degree to which scores from our test correlated expectedly with a network of previously validated measures [30]. Herein, three variables were tested as connected with the STE construct: work engagement, the type of training methods and techniques (as antecedents), and the perceived quality and usefulness of safety training in terms of safety performance (as an outcome). The conservation of resources theory [31] suggests a relationship between work engagement and STE. COR theory asserts that people strive to gain, retain, and enhance valuable resources to protect themselves from psychological harm, and achieve the desired goals. As such, people must invest resources to preserve existing resources and activate resource gain spirals. Those who dispose of resources can invest them and better sustain the resource acquisition process. Work engagement is typically seen in the literature as the outcome of an investment of personal and job resources [22,32]. However, some evidence shows that it may also represent the initiator of a motivational investment process toward resource acquisition [33,34]. In other words, work engagement may predict STE positively. Those engaged may seek to reinforce their resource acquisition and consider safety training an investment towards better skills for safety performance, increasing their cognitive involvement with the training. A similar idea is expressed by the model of Casey and colleagues [27], which lists work engagement among the pre-training factors that can influence training engagement.

Therefore, this study hypothesizes:

**Hypothesis** **1.** *Work engagement is positively associated with STE-S*.

This study compares STE scores across trainees who attended training sessions with different engaging training methods and techniques. As outlined previously, Burke and colleagues [8], in a meta-analytic study, distinguished between high-engagement training activities that stimulate the active participation (i.e., observation of a role model, feedback, hands-on demonstrations with the active participation of the trainees), and more passive and less engaging ones (i.e., passive trainees’ attending lectures to obtain health and safety-related information). Moreover, they found that the former type of training is more effective in knowledge acquisition and transfer than the latter. This suggests that a more participative and engaging training technique may influence trainees’ engagement. Indeed, some evidence exists [35,36] that situational factors (type of content, content delivery, training environment setting, trainers’ behaviors) affect trainees’ engagement in training. In relation to this, Casey and colleagues [23] model lists the type of training method among the training factors that can influence training engagement.

A valuable contribution to external validity is establishing whether training characteristics impact STE scores. To ascertain the impact of different training methods, we tested whether there were differences in the STE in trainees attending less engaging safety training sessions (i.e., classical lectures) vs. more engaging safety training sessions (i.e., behavioral stimulations, active trainees’ participation). Therefore, the following research question is posited:

**Hypothesis** **2.** *Trainees attending a more engaging safety training session will report higher STE scores compared to trainees attending a less engaging safety training session*.

The relationship between STE and perceived quality is suggested by previous research in the field of learning and training. Evidence exists that forms of involvement (e.g., cognitive, emotional) experienced by trainees are associated with some positive outcomes in terms of subjective reactions of the trainees towards training quality, such as perceived usefulness [37,38,39]

**Hypothesis** **3.** *STE-S is positively associated with the perceived usefulness of safety training*.

**Figure 1:**
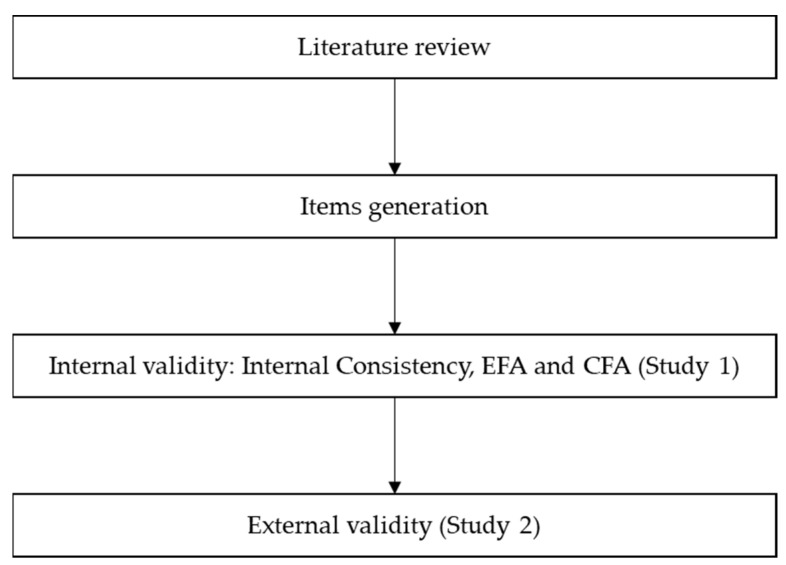
STE scale development and validation process.

The two studies conducted are presented below to describe the development and construct validity evaluation (in line with the recommendations of Grimm and Widaman, [30]) of the newly developed STE-S scale. The first study pertains to the development of the items for the scale. Moreover, this study seeks initial evidence of the scale construct (internal) validity in terms of the dimensional structure of the scale and internal consistency. The second study seeks to provide further evidence for construct validity by testing the external validity in terms of criterion-related validity. Figure 1 displays a synthesis of the process for the STE scale development.

## 2. Study 1—Items Generation and Internal Validity

### 2.1. Methods

Inductive and deductive approaches [40] were adopted to capture the features of the STE construct with a set of items. Subsequently, the dimensionality of the STE scale items was evaluated with Exploratory (EFA) and confirmatory (CFA) factor analyses with samples of safety training trainees (See Table 1 for a complete description of the participants’ profiles for EFA and CFA). 

Concerning item generation, on the basis of the literature (e.g., [26,28]), the first and the third authors of the present paper built eight items. Subsequently, three experts in safety training chose the best five items based on relevance, clarity, simplicity, and ambiguity, following the standard of the content validity [41]. Items number 1, 3, and 5 (Table 2) focus on the affective state, substantially considering the involvement and the flow of the time, and items number 2 and 3 on the cognitive state, considering the trainee’s interest and concentration. Each item invited respondents to rate their agreement on the extent to which a safety training course was involving and engaging (i.e., “think about the training course about safety in your work that you have attended, and answer the following questions.”), on a Likert scale ranging from 1 = *Not at all* to 5 = *A lot*. Item examples are: “Overall, how much did you feel involved during the course?” or “Overall, how engaging was the course you have attended?” (the eight items are listed in Table 2). 

Concerning EFA and CFA, a total of 913 participants of safety training programs in the field of the Italian chemical industry were invited to complete the five items of the STE scale after attending a safety training course between 2017–2019. The lessons concerned the non-technical skill (i.e., social and personal skills, complementary to technical skills, which contribute to safe and efficient performance) [42,43]. Moreover, the training methods were moderately engaging (i.e., feedback interventions, performance information provided in small groups) and highly engaging (i.e., behavioural stimulations that needed active participation of the trainee), following the classification of Burke and colleagues [11].

Participation was anonymous and voluntary upon informed consent from the participants. No material incentive was given. The procedures complied with the Ethical Guidelines of the Helsinki Declaration and the Italian deontological code of psychologists. All of the participants freely agreed to take part in the study, had the possibility to leave the study at any time and without compensation; the data have been analyzed, ensuring the anonymity of participants following Italian privacy law.

The majority of the sample are men (*n* = 823; 90.1%), and the mean age is 43.91 (range = 18–68) years, with a standard deviation of 10.66. The sample was split in two to run EFA and CFA. Data from a sample of 456 employees were used for EFA. Most participants were men (*n* = 417; 91.4%), and with a mean age of 41.59 years (range = 18–60; *SD* = 10.8). Subsequently, the dimensional structure of the STE scale was evaluated at a confirmatory level. A sample of 457 employees was involved. Most participants were men (*n* = 406; 88.8%), and with a mean age of 46.23 years (range = 20–68; *SD* = 10.01). 

Concerning the strategy for data analysis, for the EFA, a principal axis factoring with varimax rotation was used to select factors to retain. Items with a minimum factor loading of 0.32 were retained [44]. The CFA was performed using the AMOS software [45]. Different indices tested the model [46], namely the comparative fit index (CFI), the non-normed fit index (NNFI), the root-mean-square error of approximation (RMSEA), and the standardized root-mean-square residual (SRMR). General recommendations are that a CFI and an NNFI greater than 0.90, with RMSEA and the SRMR below 0.08, suggest a good fit [47]. At this stage, the internal consistency of the scale was evaluated (see Table 3). In line with Hair et al. [48], Cronbach’s alpha and the omega (ω) index were used. Moreover, the information derived from the CFA allowed the calculation of the composite reliability (CR) and average variance extracted (AVE) of the scale, which could be used as indicators of a scale’s internal consistency [48].

### 2.2. Results

For the EFA, a single factor is retained, accounting for 74.22% of the total variance (see Table 1 for means, standard deviations, and factor loadings of the scale). The CFA confirms the single-factor solution, and results in good fit indices (CFI = 0.99; NNFI = 0.99; RMSEA = 0.04; SRMR = 0.01). Table 2 reports the standardized factor loadings, the Cronbach’s alpha and omega values, and the CR and AVE values. Internal consistency evidence is achieved, given that the values of the indices are above the recommended threshold [31].

## 3. Study 2—External Validity

### 3.1. Methods

A total of 134 workers of a large chemical multinational company, coming from plants located in the north of Italy, were invited to complete the five items of the STE scale after attending a safety training course in 2019. 

No material incentive was given. The procedures complied with the Ethical Guidelines of the Helsinki Declaration and the Italian deontological code of psychologists. All of the participants freely agreed to take part in the study, had the possibility to leave the study at any time and without compensation; the data have been analyzed, ensuring the anonymity of participants following Italian privacy law. 

The majority of the sample were men (men = 106; 79.1%; women = 28; 20.9%), and the mean age is 45.17 (range = 25–60) years, with a standard deviation of 8.85. The majority of the participants had a high school diploma (primary education = 2, 1.5%; secondary lower education = 31, 23.1%; secondary upper education = 72, 53.7%; tertiary education = 29, 21.6%).

Concerning the measures used, STE was assessed with the same five-item scale as in the previous study. Work engagement was measured with the Italian version [49] of the Utrecht work engagement scale [50]. The scale consists of three subscales tapping the three core dimensions of work engagement: vigor (e.g., “At work, I feel bursting with energy”), absorption (e.g., “I am immersed in my job”), and dedication (e.g., “I am enthusiastic about my job”). Each subscale has three items, with a seven-point frequency scale, ranging from 0 = never to 6 = always. The Italian validation of the scale provided evidence for construct validity, such as excellent internal consistency, confirmation of the factorial structure and measurement invariance across groups, and convergent and criterion validity.

The perceived usefulness of the safety training program was measured with four items of a subscale of the work safety scale developed by Hayes and colleagues [51]. The items assessed the trainees’ reactions towards safety training with items such as: “the safety training program has been useful” or “the safety program will be effective to prevent accidents”. Items were scored on a five-point Likert scale ranging from 1 = *strongly disagree* to 5 = *strongly agree*. The original items presented excellent internal consistency (Cronbach’s alpha value = 0.93).

Two hierarchical regressions were tested. The first hierarchical regression tested STE as a dependent variable. The first step included the participants’ education level as a control variable. The second step included work engagement as the independent variable. The second hierarchical regression had the perceived usefulness of safety training as the dependent variable. The first step included the same control variable as the first hierarchical regression. The second step included STE as the independent variable.

Moreover, to test hypothesis 2, a sample of 450 workers from different Italian companies in the field of the chemical industry were invited to complete the five items of the STE scale after attending a safety training course. Participation was anonymous and voluntary upon informed consent from the participants. No material incentive was given. The procedures complied with the ethical guidelines of the Helsinki Declaration. Some of the trainees in this sample (sample A; *n* = 277) attended a different training session from the rest (sample B; *n* = 173). The majority of sample A were men (men = 241; 87%; women = 36; 13%), and the mean age was 45.3 years (range = 20–68), with a standard deviation of 10.20. The majority of the participants had a high school diploma (primary education = 6, 2.2%; secondary lower education = 97, 35%; secondary upper education = 135, 48.%; tertiary education = 39, 14.1%). The majority of sample B were men (men = 160; 92.4%; women = 13; 7.6%), and the mean age was 43.85 (range = 18–68) years, with a standard deviation of 11.78. The majority of the participants had a high school diploma (primary education = 3, 1.8%; secondary lower education = 56, 32.3%; secondary upper education = 90, 52.1%; tertiary education = 25, 14.5%).

The training sessions differed by trainees, training contents, and delivery mode of the contents. The first group (sample A) attended lessons where mainly highly engaging methods were adopted, in line with the classification of Burke and colleagues [11] (i.e., behavioural stimulation and active discussions in the class). The second group (sample B) consisted of trainees who attended lessons with less engaging methods (i.e., traditional teaching style lectures). An ANOVA was carried out to identify potential differences in STE scores among trainees attending different sessions, with respect to the method engagement.

### 3.2. Results

Table 4 shows means, standard deviations, internal consistency, and bivariate correlations among study variables. The first hierarchical regression (Table 5) reveals that education level does not influence STE, and the 1.8% variation is not significant *F*(1, 132) = 2.38, *p* = 0.13. The introduction of work engagement as the independent variable explains an additional 3.7% of the variance in STE, and this change is significant, *F*(1, 131) = 5.17, *p* < 0.05, with work engagement positively associated with STE. The evidence substantially confirms hypothesis 1. The second hierarchical regression (Table 6) reveals that none of the control variables influence perceived usefulness, and the 2.8% variation is not significant, *F*(1, 132) = 3.77, *p* = 0.05. The introduction of STE as the independent variable explains an additional 31.3% of the variance in perceived usefulness. This change is significant, *F*(1, 131) = 62.27, *p* < 0.001, with STE positively associated with perceived usefulness. This latest result confirms hypothesis 3.

Regarding hypothesis 2, a one-way ANOVA was performed to compare the effect of two different types of training methods on STE scores. The one-way ANOVA reveals that there is a statistically significant difference in mean STE scores between the two groups (F(1, 449) = 32.86, MSE = 16.67, *p* < 0.001, η^2^ = 0.07). Scores on the STE are higher among participants who attended the session with engaging methods (M = 4.2, SD = 0.61) than the score of the training session with no engaging method (M = 3.8, SD = 0.84). These results allow the achievement of external (criterion-related) validity for the newly developed STE scale.

## 4. Discussion

This research contributes to the literature by progressing the understanding of engagement in safety training programs. We present a new way to assess the construct based on the lack of reliable and validated tools to measure training engagement. On a theoretical level, this study expands the knowledge about engagement in safety training programs by posing from a trainee’s perspective. We, therefore, present a scale of measure, namely STE-S, which presents a mono-dimensional model. The studies of STE-S development and validation demonstrate satisfactory results. Regarding internal validity, in terms of dimensionality, the EFA (Study 1) suggests the existence of a one-factor structure of the STE-S construct. CFA shows a good fit for the one-factor model, and confirms the structure and dimensionality in the subsequent analysis.

Findings regarding the external validity (Study 2) are reasonable and in line with the hypotheses. Work engagement predicts, as expected, the STE-S. The effect is not high, but significant at an alpha level of 0.05, confirming hypothesis 1. This aligns with the COR theory framework [31] and the idea that work engagement, rather than only being an outcome of resource investment and acquisition, is also an initiator of the resource gain process. Engagement motivates investment in training to acquire more skills for better safety performance, increasing cognitive involvement with the training [33,34]. Moreover, results show a significant difference between STE scores of those attending more engaging training vs those attending less engaging training sessions, confirming hypothesis 2.

Finally, the results show that STE-S predicts the perceived usefulness of safety training. In this case, the effect is not only significant, but high too. This confirms hypothesis 3, aligning with the existent evidence that the attendee’s forms of involvement (e.g., cognitive, emotional) predict positive reactions to the training quality, such as perceived usefulness [37,38,39].

The development and validation of the STE-S scale have several strengths, with theoretical and practical implications. First, this scale’s development and validation processes adhere to a thorough review of the theoretical and empirical literature on training and safety training. This allows us to contribute to the conceptualization of STE constructs, and build a scale with good evidence of internal validity, considering reliability and dimensionality [30]. Second, the external validity analyses present good results. The impact of work engagement on STE scores aligns with the COR theory reasoning that resourceful people (i.e., with higher levels of work engagement) may want to reinforce their work-related resource pool. In the specific case of this study, more engaged people may consider safety training as a means to gain further resources and improve their work safety performances, which higher levels of STE may reflect. The influence of STE on the perceived usefulness of the safety training also follows the existing literature, corroborating that cognitive and emotive involvement in training may enhance trainees’ subjective reactions towards training. Furthermore, the differences found in STE scores depending on less versus more engaging training methods are valuable to the literature. Indeed, beyond the positive impact of more trainee-centered, engaging training techniques on training effectiveness (e.g., knowledge acquisition and transfer; 8), this study also shows that more engaging methods influence trainees’ engagement. Future research should unravel the relationships linking training methods, trainees’ engagement, and training effectiveness.

At a practical level, this study delivers an agile tool to use both in research and intervention settings, because it is easy and fast to use. STE-S may help scholarly work in extending knowledge about the network of psychosocial factors involved in safety training effectiveness. On the other hand, safety trainers can monitor trainees’ engagement and, consequently, be able to adjust the training contents or methods. Even though the STE-S scale does not allow an analytic measure of the different components of engagement (affective, cognitive, and behavioral) as suggested by Ben-Eliyahu and colleagues [16], its brevity (5 items, in contrast to the 17 items of the Ben-Eliyahu and colleagues scale in [16]), makes it an agile tool in application contexts. For instance, it can also be used in interactive presentation tools (e.g., the online tools or sevices of Mentimeter ©, Slido ©), which are, nowadays, increasingly adopted in both face-to-face and remote training, particularly used in post-COVID periods, or with workers with low education levels, as is sometimes found among migrants in the construction industry [7], where it is necessary to use an easy and not very complex questionnaire.

This study has some limitations. First, the samples involved only workers of chemical plants that attended courses on non-technical skills, as described in the methodological section. Such homogeneous samples may have some drawbacks in terms of external validity. Concerning the external validity, we advocate for further research to address the convergent and discriminant validity of the STE-S. Moreover, the cross-sectional design used herein does not make it possible to infer causal relationships among the study variables, thus, reducing our results’ strength in predictive validity. Lastly, it should be noted that the items on the scale have non-variegated and tendentially low difficulty values. However, regarding the first aspect, the scale has a good internal homogeneity, and evidence of a consistent variability between the indicators. Concerning the second aspect, we believe that it is necessary to adopt wider samples that consider courses of different nature and structure for a more in-depth evaluation.

## 5. Conclusions

The study makes a significant attempt at conceptualizing and developing the STE construct from a subjective and individual perspective. A newly developed short measure of STE (i.e., STE-S) is coherently presented, and an initial validation is performed in terms of internal and external validity, with Italian employees working in chemical companies and attending safety training courses. In study 1, a single-factor structure of the STE construct emerges from the EFA and CFA, and good internal consistency indicators values are achieved. Study 2 draws on the existing literature to reach evidence of the external validity of the scale, showing that: (1) work engagement predicts the STE scale; (2) trainees of courses where participative/engaging methods are adopted present a higher level of STE compared to trainees of courses where there are no participative/engaging methods; and (3) the STE score predicts the perceived usefulness of the safety training. Although further investigations are needed to increase the robustness of the STE-S’s validity, the use of such a scale may present both research and practice with significant benefits in monitoring the trainees’ level of engagement in a safety course, with a very brief measure that shows the engagement and involvement of trainees.

## Figures and Tables

**Table 1:** Characteristics of the sample for EFA and CFA.

Demographic Variables				
	EFA		CFA	
	*n*	%	*n*	%
Gender				
Man	417	91.4	406	88.8
Woman	39	8.6	51	11.2
Age				
18–29	98	21.5	47	10.3
30–39	65	14.3	54	11.8
40–49	162	35.5	149	32.6
50–59	129	28.3	188	41.2
60–68	2	0.4	19	4.2
Educational Level				
Primary education	4	0.9	8	1.8
Lower secondary education	150	32.9	138	30.2
Upper secondary education	221	48.5	242	53.0
Tertiary education	81	17.8	53	11.6
Type of Contract				
Part-time permanent contract	7	1.5	3	.7
Full-time permanent contract	348	76.3	409	89.5
Part-time temporary contract	1	.2	4	0.9
Full-time temporary contract	59	12.9	22	4.8
Other	41	9.0	19	4.2

**Table 2:** Means, standard deviations, and factor loadings of the STE items.

	Items	*M*	*SD*	Factor Loadings
*“Think about the training course about safety in your work that you have attended, and answer the following questions.”*	1. En. Overall, how engaging was the training course you attended?	4.08	0.81	0.85
	It. Complessivamente quanto è stato coinvolgente il corso al quale ha partecipato?			
	2. En. Overall, how interesting was the training course you attended?	4.07	0.82	0.85
	It. Complessivamente quanto è stato coinvolgente il corso al quale ha partecipato?			
	3. En. Overall, how involved did you feel during the training course?	4.11	0.79	0.82
	It. Complessivamente quanto si è sentito coinvolto durante il corso?			
	4. En. Overall, was it easy to maintain concentration during the training course you attended?	3.93	0.87	0.79
	It. Complessivamente è stato facile mantenere la concentrazione durante il corso al quale ha partecipato?			
	5. En. Time flew by during the training course.	3.82	0.97	0.79
	It. Il tempo è volato durante il corso di formazione.			

*Note. n =* 456. It = Italian version, En = English translation.

**Table 3:** CFA standardized factor loadings and internal consistency values of the STE scale.

	Items	λ	α	ω	CR	AVE
*“Think about the training course about safety in your work that you have attended, and answer the following questions.”*	En. Overall, how engaging was the training course you attended?	0.92	0.93	0.93	0.92	0.72
	It. Complessivamente quanto è stato coinvolgente il corso al quale ha partecipato?					
	2. En. Overall, how interesting was the training course you attended?	0.91				
	It. Complessivamente quanto è stato interessante il corso al quale ha partecipato?					
	3. En. Overall, how involved did you feel during the training course?	0.85				
	It. Complessivamente quanto si è sentito coinvolto durante il corso?					
	4. En. Overall, was it easy to maintain concentration during the training course you attended?	0.76				
	It. Complessivamente è stato facile mantenere la concentrazione durante il corso al quale ha partecipato?					
	5. En. Time flew by during the training course.	0.77				
	It. Il tempo è volato durante il corso di formazione.					

*Note. n* = 456. It = Italian version, En = English translation. λ = standardized factor loadings; α = Cronbach’s alpha value, ω = omega value, CR = composite reliability, AVE = average variance extracted.

**Table 4:** Means, standard deviations, internal consistency, and bivariate correlations among study variables.

Variable	M (SD)	α	1.	2.	3.
Educational level ^a^					
2. Work engagement	4.69 (0.89)	0.87	0.07		
3. STE	4.20 (0.79)	0.92	−0.13	0.18 *	
4. Perceived Usefulness	3.91 (0.75)	0.89	−0.17	0.17	0.58 **

*Note. n* = 134. ^a^ 1 = primary school diploma, 2 = secondary school diploma, 3 = high school diploma, 4 = university degree, 5 = post-graduation degree. STE = safety training engagement. α = Cronbach’s alpha value. * *p* < 0.01; ** *p* < 0.05.

**Table 5:** Hierarchical regression analysis for nomological validity (STE as dependent variable).

Variable	R^2^	ΔR^2^	B	β	*SE*	*t*-Value	*p*
Step 1	0.02						
Educational level ^a^			−0.13	−0.13	0.08	−1.54	0.13
Step 2	0.08	0.04					
Educational level ^a^			−0.14	−0.15	0.08	−1.71	0.09
Work engagement			0.17	0.19	0.08	2.27	0.03

*Note. n* = 133. ^a^ 1 = primary school diploma, 2 = secondary school diploma, 3 = high school diploma, 4 = university degree, 5 = post-graduation degree.

**Table 6:** Hierarchical regression analysis for nomological validity (STE as the independent variable).

Variable	R^2^	ΔR^2^	B	β	*SE*	*t*-Value	*p*
Step 1	0.03						
Educational level ^a^			−0.15	−0.17	0.08	−1.94	0.05
Step 2	0.34	0.31					
Educational level ^a^			−0.08	−0.09	0.07	−1.28	0.20
STE			0.54	0.57	0.07	7.89	0.000

*Note. n* = 133. ^a^ 1 = primary school diploma, 2 = secondary school diploma, 3 = high school diploma, 4 = university degree, 5 = post-graduation degree. STE = safety training engagement.

## Data Availability

The data that support the findings of this study are available upon reasonable request from the corresponding author. The data are not publicly available due to restrictions from the companies that were involved in this study.

## References

[1] Eurostat Accidents at Work Statistics. https://ec.europa.eu/eurostat/statistics-explained/index.php/Accidents_at_work_statistics.

[2] Ingram C., Downey V., Roe M., Chen Y., Archibald M., Kallas K.A., Kumar J., Naughton P., Uteh C.O., Rojas-Chaves A. (2021). COVID-19 Prevention and Control Measures in Workplace Settings: A Rapid Review and Meta-Analysis. Int. J. Environ. Res. Public Health.

[3] Komol M.M.R., Hasan M.M., Elhenawy M., Yasmin S., Masoud M., Rakotonirainy A. (2021). Crash severity analysis of vulnerable road users using machine learning. PLoS ONE.

[4] Kariuki S.G., Löwe K. (2007). Integrating human factors into process hazard analysis. Reliab. Eng. Syst. Saf..

[5] Ren J., Jenkinson I., Wang J., Xu D.L., Yang J.B. (2008). A methodology to model causal relationships on offshore safety assessment focusing on human and organizational factors. J. Saf. Res..

[6] Sexton J.B., Helmreich R.L. (2000). Analyzing Cockpit Communications: The Links Between Language, Performance, Error, and Workload. J. Hum. Perform. Extrem. Environ..

[7] Peiró J.M., Nielsen K., Latorre F., Shepherd R., Vignoli M. (2020). Safety training for migrant workers in the construction industry: A systematic review and future research agenda. J. Occup. Health Psychol..

[8] Valori M., Scibilia A., Fassi I., Saenz J., Behrens R., Herbster S., Bidard C., Lucet E., Magisson A., Schaake L. (2021). Validating Safety in Human–Robot Collaboration: Standards and New Perspectives. Robotics.

[9] Magnavita N., Soave P.M., Antonelli M. (2021). Teaching safety—Resident anaesthetists at the forefront of COVID-19. Ind. Health.

[10] Ricci F., Chiesi A., Bisio C., Panari C., Pelosi A. (2016). Effectiveness of occupational health and safety training: A systematic review with meta-analysis. J. Workplace Learn..

[11] Burke M.J., Sarpy S.A., Smith-Crowe K., Chan-Serafin S., Salvador R.O., Islam G. (2006). Relative effectiveness of worker safety and health training methods. Am. J. Public Health.

[12] Morgan R.B., Casper W.J. (2000). Examining the factor structure of participant reactions to training: A multidimensional approach. Hum. Resour. Dev. Q..

[13] Ritzmann S., Hagemann V., Kluge A. (2014). The Training Evaluation Inventory (TEI)—Evaluation of Training Design and Measurement of Training Outcomes for Predicting Training Success. Vocat. Learn..

[14] Grohmann A., Kauffeld S. (2013). Evaluating training programs: Development and correlates of the Questionnaire for Professional Training Evaluation. Int. J. Train. Dev..

[15] O’Brien H.L., Toms E.G. (2010). The development and evaluation of a survey to measure user engagement. J. Am. Soc. Inf. Sci. Technol..

[16] Ben-Eliyahu A., Moore D., Dorph R., Schunn C.D. (2018). Investigating the multidimensionality of engagement: Affective, behavioral, and cognitive engagement across science activities and contexts. Contemp. Educ. Psychol..

[17] Robson L.S., Stephenson C.M., Schulte P.A., Amick B.C., Irvin E.L., Eggerth D.E., Chan S., Bielecky A.R., Wang A.M., Heidotting T.L. (2012). A systematic review of the effectiveness of occupational health and safety training. Scand. J. Work Environ. Health.

[18] Mariani M.G., Curcuruto M., Matic M., Sciacovelli P., Toderi S. (2017). Can leader-member exchange contribute to safety performance in an Italian warehouse?. Front. Psychol..

[19] Namian M., Albert A., Zuluaga C.M., Jaselskis E.J. (2016). Improving Hazard-Recognition Performance and Safety Training Outcomes: Integrating Strategies for Training Transfer. J. Constr. Eng. Manag..

[20] Burke M.J., Salvador R.O., Smith-Crowe K., Chan-Serafin S., Smith A., Sonesh S. (2011). The dread factor: How hazards and safety training influence learning and performance. J. Appl. Psychol..

[21] Luthans F. (2002). The need for and meaning of positive organizational behavior. J. Organ. Behav..

[22] Mazzetti G., Robledo E., Vignoli M., Topa G., Guglielmi D., Schaufeli W.B. (2021). Work Engagement: A meta-Analysis Using the Job Demands-Resources Model. Psychol. Rep..

[23] Hallberg U.E., Schaufeli W.B. (2006). “Same Same” But Different?. Eur. Psychol..

[24] Hidi S., Renninger K.A. (2006). The Four-Phase Model of Interest Development. Educ. Psychol..

[25] Lazowski R.A., Hulleman C.S. (2016). Motivation Interventions in Education. Rev. Educ. Res..

[26] Lee J.S. (2014). The Relationship Between Student Engagement and Academic Performance: Is It a Myth or Reality?. J. Educ. Res..

[27] Casey T., Turner N., Hu X., Bancroft K. (2021). Making safety training stickier: A richer model of safety training engagement and transfer. J. Saf. Res..

[28] Schaufeli W.B., Salanova M., González-romá V., Bakker A.B. (2002). The Measurement of Engagement and Burnout: A Two Sample Confirmatory Factor Analytic Approach. J. Happiness Stud..

[29] Aguinis H., Kraiger K. (2009). Benefits of Training and Development for Individuals and Teams, Organizations, and Society. Annu. Rev. Psychol..

[30] Grimm K.J., Widaman K.F. (2012). Construct validity. APA Handbook of Research Methods in Psychology, Vol 1: Foundations, Planning, Measures, and Psychometrics.

[31] Hobfoll S.E., Halbesleben J., Neveu J.P., Westman M. (2018). Conservation of Resources in the Organizational Context: The Reality of Resources and Their Consequences. Annu. Rev. Organ. Psychol. Organ. Behav..

[32] Mazzetti G., Vignoli M., Petruzziello G., Palareti L. (2019). The Hardier You Are, the Healthier You Become. May Hardiness and Engagement Explain the Relationship Between Leadership and Employees’ Health?. Front. Psychol..

[33] Laguna M., Razmus W. (2019). When I Feel My Business Succeeds, I Flourish: Reciprocal Relationships Between Positive Orientation, Work Engagement, and Entrepreneurial Success. J. Happiness Stud..

[34] Bakker A.B., Bal M.P. (2010). Weekly work engagement and performance: A study among starting teachers. J. Occup. Organ. Psychol..

[35] Kim S., Park S., Lavelle J., Kim M., Chaudhuri S. (2020). Revisiting trainee reactions: A multilevel analysis of the nomological network. Hum. Resour. Dev. Q..

[36] Brown K.G. (2005). An Examination of the Structure and Nomological Network of Trainee Reactions: A Closer Look at “Smile Sheets”. J. Appl. Psychol..

[37] Tracey J.B., Hinkin T.R., Tannenbaum S., Mathieu J.E. (2001). Characteristics and the Work Environment on Varying Levels of Training Outcomes. Hum. Resour. Dev. Q..

[38] Reychav I., Wu D. (2015). Are your users actively involved? A cognitive absorption perspective in mobile training. Comput. Hum. Behav..

[39] Ginting H., Mahiranissa A., Bekti R., Febriansyah H. (2020). The effect of outing Team Building training on soft skills among MBA students. Int. J. Manag. Educ..

[40] MacKenzie S.B., Podsakoff P.M., Podsakoff N.P. (2011). Construct Measurement and Validation Procedures in MIS and Behavioral Research: Integrating New and Existing Techniques. MIS Q..

[41] Polit D.F., Beck C.T., Owen S.V. (2007). Is the CVI an acceptable indicator of content validity? Appraisal and recommendations. Res. Nurs. Health.

[42] Flin R., O’Connor P., Crichton M. (2008). Safety at the Sharp End: A Guide to Non-Technical Skills.

[43] Mariani M., Vignoli M., Chiesa R., Violante F., Guglielmi D. (2019). Improving Safety through Non-Technical Skills in Chemical Plants: The Validity of a Questionnaire for the Self-Assessment of Workers. Int. J. Environ. Res. Public Health.

[44] Worthington R.L., Whittaker T.A. (2006). Scale Development Research. Couns. Psychol..

[45] Arbuckle J.L. (2012). IBM SPSS Amos 23 User’s Guide.

[46] Cheung G.W., Rensvold R.B. (2002). Evaluating Goodness-of-Fit Indexes for Testing Measurement Invariance. Struct. Equ. Modeling A Multidiscip. J..

[47] Hu L., Bentler P.M. (1999). Cutoff criteria for fit indexes in covariance structure analysis: Conventional criteria versus new alternatives. Struct. Equ. Modeling A Multidiscip. J..

[48] Hair J.F., Babin B.J., Anderson R.E., Black W.C. (2019). Multivariate Data Analysis.

[49] Balducci C., Fraccaroli F., Schaufeli W.B. (2010). Psychometric Properties of the Italian Version of the Utrecht Work Engagement Scale (UWES-9). Eur. J. Psychol. Assess..

[50] Schaufeli W.B., Bakker A.B., Salanova M. (2006). The Measurement of Work Engagement With a Short Questionnaire. Educ. Psychol. Meas..

[51] Hayes B.E., Perander J., Smecko T., Trask J. (1998). Measuring Perceptions of Workplace Safety. J. Saf. Res..

